# Cations Modulated Assembly of Triol-Ligand Modified Cu-Centered Anderson-Evans Polyanions

**DOI:** 10.3390/molecules27092933

**Published:** 2022-05-04

**Authors:** Yiran Wang, Fengxue Duan, Xiaoting Liu, Bao Li

**Affiliations:** State Key Laboratory of Supramolecular Structure and Materials, College of Chemistry, Jilin University, Changchun 130012, China; wangyr19@mails.jlu.edu.cn (Y.W.); duanfx17@mails.jlu.edu.cn (F.D.); liuxt16@mails.jlu.edu.cn (X.L.)

**Keywords:** polyoxometalate, Anderson-Evans, triol ligand, cation-modulation

## Abstract

Counter-cations are essential components of polyoxometalates (POMs), which have a distinct influence on the solubility, stabilization, self-assembly, and functionality of POMs. To investigate the roles of cations in the packing of POMs, as a systematic investigation, herein, a series of triol-ligand covalently modified Cu-centered Anderson-Evans POMs with different counter ions were prepared in an aqueous solution and characterized by various techniques including single-crystal X-ray diffraction. Using the strategy of controlling Mo sources, in the presence of triol ligand, NH_4_^+^, Cu^2+^ and Na^+^ were introduced successfully into POMs. When (NH_4_)_6_Mo_7_O_24_ was selected, the counter cations of the produced POMs were ammonium ions, which resulted in the existence of clusters in the discrete state. Additionally, with the modulation of the pH of the solutions, the modified sites of triol ligands on the cluster can be controlled to form δ- or χ-isomers. By applying MoO_3_ in the same reaction, Cu^2+^ ions served as linkers to connect triol-ligand modified polyanions into chains. When Na_4_Mo_8_O_26_ was employed as the Mo source to react with triol ligands in the presence of CuCl_2_, two 2-D networks were obtained with {Na_4_(H_2_O)_14_} or {{Na_2_(H_2_O)_4_} sub-clusters as linkers, where the building blocks were δ/δ- and χ/χ-isomers, respectively. The present investigation reveals that the charges, sizes and coordination manners of the counter cations have an obvious influence on the assembled structure of polyanions.

## 1. Introduction

As an important and basic type of polyoxometalates (POMs), Anderson-Evans clusters have been synthesized and characterized for nearly one hundred years, with a general formula of [X^n+^M_6_O_24_H_m_]^(12−n−m)−^, in which X expresses the heteroatom and M the addenda atoms (Mo or W in most cases) [1,2,3]. Compared with the terminal O atoms in Anderson-Evans polyanion, the bi- or tri-bridging O atoms (μ_2_- or μ_3_-O) have a higher reactive activity and can be replaced by some organic species with hydroxyl groups under specific circumstances to form various decoration types (Figure S1, Supplementary Materials) [4,5,6]. Through this method, different organic functional groups can be introduced into the inorganic skeleton, which not only enriches the structural figures of the final adducts but also integrates the properties of both parts, resulting in novel functionalities [7,8,9,10,11]. Because the organic functional groups have different hydrophilic and hydrophobic properties, they need to be introduced into polyanions under different solvent environments, such as water or organic solvents [12,13], while due to the use of TBA_4_Mo_8_O_26_ (TBA = tetrabutylammonium cation) as the Mo source in most cases, triol-ligand modified Anderson-Evans polyanions are mostly prepared in organic solvents such as CH_3_CN, CH_3_OH and *N*,*N*-dimethylformamide, which limits their applications [14,15]. So, it is necessary to find new Mo sources, for example Na_2_MoO_4_, (NH_4_)_6_Mo_7_O_24_, and Na_4_Mo_8_O_26_, suitable for reactions in aqueous solution, which may also bring new architectures. On the other hand, compared with the wide range of investigations on organic components covalently modified Anderson-Evans polyanions with trivalent heteroatoms, examples with divalent center atoms are less focused on and reported [16,17,18]. Aside from the initial instances with Zn^2+^ and Ni^2+^ used as heteroatoms [19], investigations on the triol-ligand covalently modified Cu^II^-, Co^II^- and Ni ^II^-centered Anderson-Evans polyanions are conducted, including finding new modification types, their transformation between different modification architectures and the co-modification of triol ligand and methanol or acetic acid [20,21,22,23,24].

Except developing new modification manners for discrete polyanions, it is also important to construct extended structures by employing the triol-ligand modified Anderson-Evans polyanions as building blocks in appropriate ways. As a comparison, one dimensional (1D) to three dimensional (3D) structures based on undecorated Anderson-Evans polyanions have been reported, in which terminal O atoms of the clusters are used to coordinate with transition metal ions for the formation of the extended organic-inorganic hybrids [25,26,27,28]. For the triol-ligand decorated Anderson-Evans polyanions, only a few cases have been reported. For example, through the introduction of pyridine groups, the discrete polyanions can be assembled into an extended structure through M–N bonds, where N atoms are sourced from the pyridine groups [29,30,31,32]. Another important strategy is linking amino functionalized Anderson-Evans clusters with 4-connected building units though imine condensation to form metal–organic frameworks [33,34]. As a more common method, rare earth ions (Ln^3+^) can also be used as nodes to link this type of cluster for the formation of coordination polymers through Ln–O bonds [35]. However, there are still no systematical investigations available on the triol-ligand modified X^II^-centered Anderson-Evans polyanions in aqueous solution, especially with regard to the influence of cations on the assembled structures of polyanions [36].

**Scheme 1:**
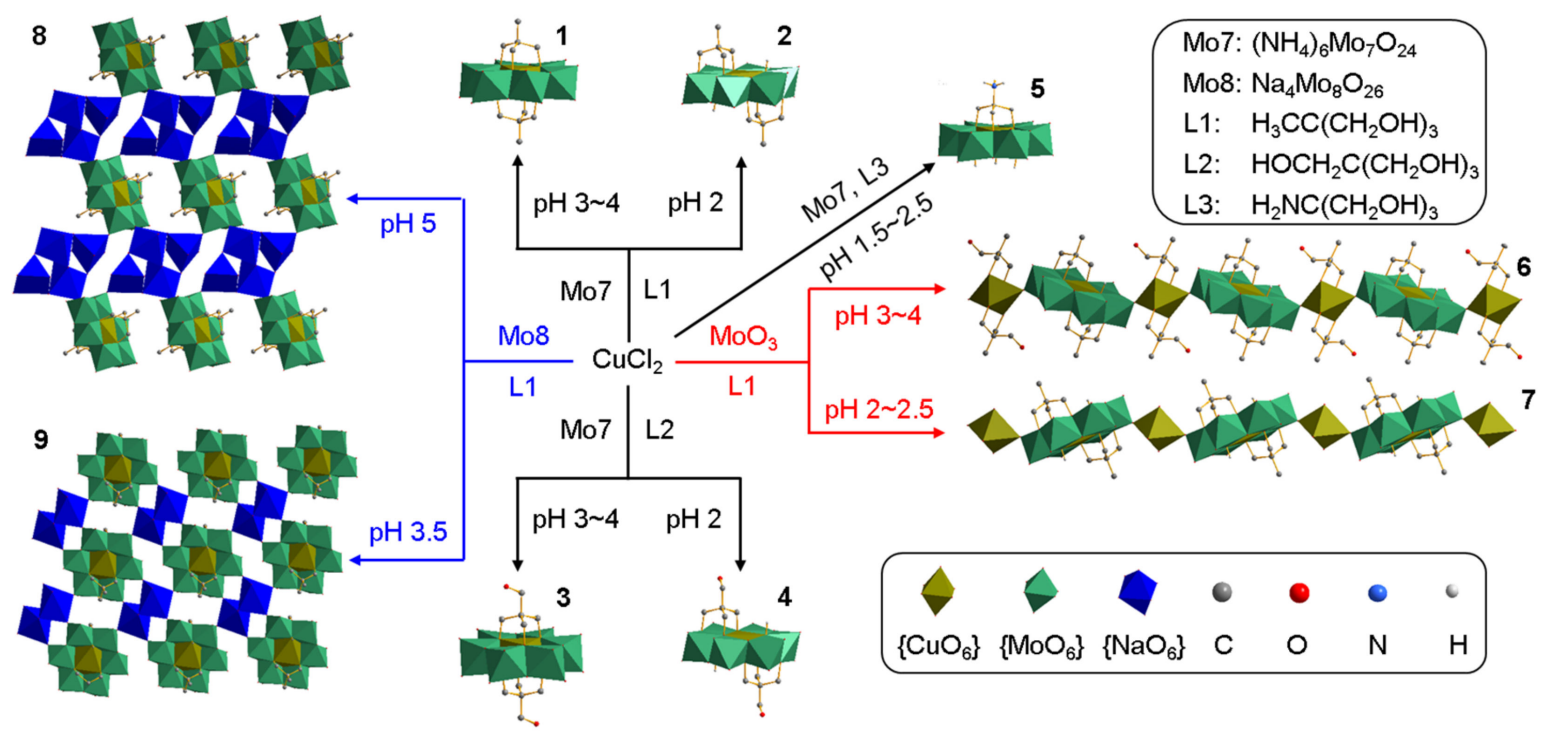
Schematic illustration for the synthetic parameters in different decoration fashions and extended structures in the preparation of Cu-centered Anderson–Evans POMs **1**–**9**. All H atoms except those attaching to N and μ_3_-O atoms are omitted for clarity. The bold numbers are added to represent the corresponding compounds.

Considering the investigations in this field, in the present contribution, we investigate the synthesis of triol-ligand modified Cu^II^-centered Anderson-Evans polyanions in detail in an aqueous solution. By selecting (NH_4_)_6_Mo_7_O_24_, MoO_3_ and Na_4_Mo_8_O_26_ as our Mo sources, which have not been widely applied before, we synthesized a series of divalent metal ion-centered clusters, and also realized the controlled modulation of an assembly of polyanions with NH_4_^+^, Cu^2+^ and Na^+^ as cations (Scheme 1). In addition, the influences of the size and connecting mode of linkers was discussed, as well as a Hirshfeld analysis of the building blocks with various decoration types. The presented results not only provide an efficient synthetic route for triol-ligand modified Anderson-Evans polyanions with divalent heteroatoms, but also express the important role of cations in the assembly of polyanions.

## 2. Results and Discussion

The prepared compounds **1**–**9** were characterized by single crystal X-ray diffraction analysis, elemental analysis, as well as IR spectra, which can be found in the Supplementary Materials as Figure S2. The thermal stability and the crystalline purity of compounds **1**–**9** were also evaluated, as shown in Figures S3–S11 and Figures S12–S14.

### 2.1. Preparation of Triol-Ligand Decorated Anderson-Evans POM Building Blocks

**Figure 1:**
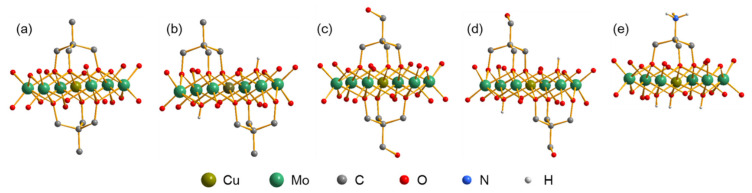
Polyanionic structures of compounds (**a**) **1**, (**b**) **2**, (**c**) **3**, (**d**) **4**, and (**e**) **5** in ball-and-stick representation. All H atoms except those attaching to N and μ_3_-O atoms are omitted for clarity.

In order to investigate the assembly behaviors of triol-ligand covalently modified Anderson-Evans polyanions in the presence of different cations, we first investigated the synthesis of their building blocks in aqueous solutions in detail. Two important factors are considered in the selection of Mo sources. The first factor is that the used Mo sources can dissolve easily in water at the initial state or in the reaction process, which ensures the occurrence of the reaction and the acceptable yields. The second controls counter cations in the final adducts through the introduction of different Mo sources. For example, (NH_4_)_6_Mo_7_O_24_ and Na_4_Mo_8_O_26_ result in NH_4_^+^ and Na^+^ as counter cations, while MoO_3_ does not lead to the formation of new metal ions, which brings about Cu^2+^ which serves as counter cation. Based on the above analysis, herein, to synthesize building blocks, (NH_4_)_6_Mo_7_O_24_ was used as an Mo source to react with CuCl_2_ and triol ligand in the aqueous solution at 80 °C. When a triol ligand with a methyl as end group was used and the pH of the solution was adjusted to 3~4, compound **1** is obtained, in which triol ligands functionalized on the Anderson-Evans polyanion in a double-sided style to form δ/δ isomer (Figure 1a). In this case, two triol ligands distributed on both sides of the Anderson-Evans polyanion, and replaced all six hydroxyls around the central heteroatom. When the pH of the solution was lowered to 2, in the case of a stronger acidic environment, partial μ_2_-O atoms were activated, and therefore each triol ligand replaced two μ_3_-O atoms and one adjacent μ_2_-O atom, resulting in a malposition modified structure compound **2** in χ/χ isomer (Figure 1b). The above results show that, at a higher pH value, triol ligands tend to replace all μ_3_-O atoms to form a δ modification style; while in a lower pH environment, triol ligands are prone to substitute partial μ_3_-O atoms, forming in an χ modification manner, where such phenomena are consistent with the literature [20]. In order to verify the universality of this method and the reliability of the conclusion, we used a triol ligand with a terminal hydroxyl group. Experiments show that, in accordance with the case of the triol ligand with the methyl group at the end, when the pH of the solution is 3~4, the hydroxyl-containing triol ligands replace all the μ_3_-O atoms to obtain a double-sided modified Anderson-Evans POM in δ/δ isomer, compound **3** (Figure 1c). When the pH value of the solution is 2, the triol ligands replace partial μ_3_-O atoms to obtain a bilaterally modified structure, compound **4**, in χ/χ isomer (Figure 1d). The above experiments show that the modified positions of the triol ligands on the Anderson-Evans polyanion can be modulated by adjusting the pH of the solution. Interestingly, it is different from those triol ligands with hydroxyl and methyl groups, when the end group is amino, we get a single-sided triol ligand decorated δ isomer, compound **5**, in the solution with a relatively lower pH of 1.5~2.5, in which adduct the amino group is in a protonated state (Figure 1e). Even the excessive triol ligands were added in this reaction, and the obtained products were still in a single-sided decoration state with the other side left free. A similar situation also exists in Al-centered Anderson-Evans POMs when modified by triol ligands in the aqueous solution [12]. When the pH of the solution rises with the amino group in the non-protonated state, the lone pair of electrons of the N atom combines easily with the d orbital of the transition metal ion Cu^2+^, which results in the crosslinking between the generated adducts and makes it difficult to obtain single crystals. An effective way to introduce a non-protonated amino-containing triol ligand into Cu-centered Anderson-Evans cluster is through a two-step synthesis procedure. That is, an undecorated Cu-centered cluster can be synthesized firstly and then used to react with the triol ligand, through which method the coordination sites of Cu^2+^ are fully occupied by O atoms, losing the combining ability with other atoms or functional groups such as amino groups. With this synthetic route, the amino-containing triol ligand can be anchored on the Cu-centered Anderson-Evans cluster in a mono-decoration type through micro-assisted synthesis [37] and double-decoration type through a regular beaker reaction in aqueous solution [38].

In all the five compounds, due to the compact and symmetric coordination environments of the Cu^2+^ ion, which is located at the center of the Anderson-Evans cluster, there is no obvious Jahn-Teller effect. Taking compound **5** as an example, the six Cu–O bond lengths were 1.958(2), 1.980(2), 1.992(2), 2.026(2), 2.195(2), and 2.213(3) Å, respectively, showing an averaged result without an extra-long Cu–O bond of over 2.4 Å. It is interesting that though half of the coordination sites of the Cu^2+^ ion were occupied by the triol ligand, the Cu–O bond lengths expressed no differences to those formed by hydroxyls. The similar coordination conditions also existed in compounds **1**–**4**.

It is worth noting that although the prepared organic–inorganic hybrids were all based on the same polyanion Cu-centered Anderson-Evans cluster, due to the different modification positions of the triol ligands on the polyanion, the charges of the obtained clusters were unequal. When the triol ligands are modified on the polyanion in δ isomer with all the hydroxyls around central heteroatom being replaced, the charge of the anion remains unchanged before and after the substitution. When the triol ligands modify in malposition on the polyanion, two unreacted hydroxyls are retained, accompanying with the replacement of two unprotonated μ_2_-O (in −2 valence) by the O atom (in −1 valence) from the hydroxyl, and resulting in a decrease in the entire anion charge from −4 to −2. When the terminal group of a triol ligand is amino, although it substitutes all protonated μ_3_-O atoms, the amino group is in a protonated state with an additional positive charge, so the charge of the entire anion is −3. That is, under different environmental conditions, we can make triol-ligand modified Cu-centered Anderson-Evans polyanions with 2~4 negative charges. This charge tunability is useful for the further development and utilization of polyanions, especially in terms of providing convenience to the controllable assembly based on the charge number.

### 2.2. Construction of 1D Structures Based on Triol-Ligand Decorated Anderson-Evans POMs

**Figure 2:**
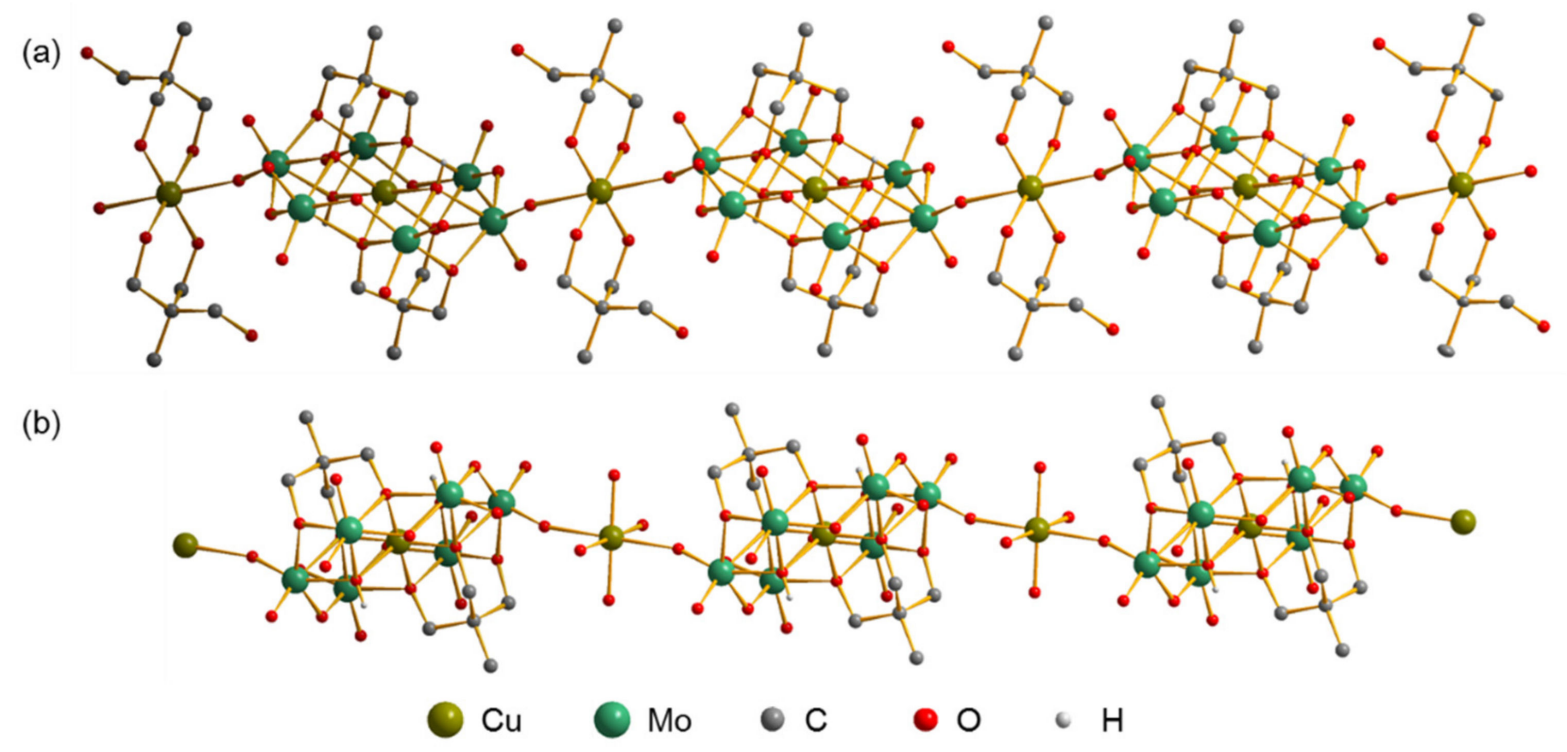
Ball-and-stick representations of compounds (**a**) **6** and (**b**) **7**, showing their 1D chain structures. All H atoms except those attaching to tri-bridging O atoms in polyanions are omitted for clarity.

After obtaining the modification law of the triol ligands on the Anderson-Evans polyanion in aqueous solution, we attempted to obtain extended structures. When ammonium is applied as counter ion, it mainly combines with anion through electrostatic interactions. The lack of directionality and selectivity of the electrostatic interactions makes it unsuitable for the ordered assembly of polyanions. Therefore, we selected MoO_3_ instead of (NH_4_)_6_Mo_7_O_24_ as the Mo source, thereby eliminating the possibility of ammonium as counter ions in the adduct. At low pH environments, two 1D chain structures with Cu^2+^ serving as linkers were obtained (Figure 2). The single-crystal X-ray diffraction results show that the Anderson-Evans polyanions were modified by triol ligands to form an χ/χ isomer, to which adjacent polyanions are further linked by Cu^2+^ through the terminal O atoms. The Cu^2+^ shows an octahedron coordination environment with an obvious Jahn-Teller effect, where two Cu–O bonds (bond lengths 2.323(2)–2.396(2) Å) connected to the polyanion are significantly elongated compared with the other four Cu–O bonds (bond length 1.924(2)–1.976(2) Å). The main difference between the two 1D compounds is that of the coordination environments of the linker Cu^2+^ ions. In compound **6**, except two terminal O atoms of clusters, four O atoms from two triol ligands complete the coordination environment of the Cu^2+^ ion; while in compound **7**, the four positions are occupied by coordinated water molecules. In compound **6**, only two hydroxyls of each triol ligand coordinate with Cu^2+^, and the other one remains in a free state. Not only coordinated hydroxyls but also free hydroxyls are in the protonated state. In the two compounds, the linker Cu^2+^ ions have different coordination environments, and the reason is that at the environment of pH 3~4, although the hydroxyls are in a protonated state, they still have a certain coordination ability with the Cu^2+^ ion, and thus occupy the four coordination sites to obtain compound **6**. However, as the pH value decreases to 2~2.5, the interaction between the hydroxyls and the Cu^2+^ ion weakens to diminish the coordination ability, so that the water molecules occupy the corresponding coordination sites, resulting in compound **7**. This statement can be verified in experiments in which the aqueous solution of compound **6** was acidified and recrystallized to obtain compound **7**. The differences in the coordination modes of the Cu^2+^ ions in the two compounds also have a certain effect on its extending direction. Compound **6** stretches along the (111) direction, while the direction of the 1D chain in compound **7** is (100).

In the two compounds, the charges of the polyanion and copper ion were −2 and +2, respectively. According to the theory of electrical neutrality, the two components are more easily combined in a 1:1 manner to form the 1D chain structure or the two-dimensional (2D) planar structure in crystallography, as shown in Figure S15, which has a relative low energy and is more stable. In the present case, because the coordination radius of the Cu^2+^ ion was not large enough, and four polyanions cannot be uniformly arranged around one Cu^2+^ ion due to the large steric hindrance, only a 1D chain structure was formed. On another hand, due to the low pH value of the solution, in both 1D structures, the triol ligands were modified on the polyanion in the χ/χ isomer. In order to obtain 1D structures based on the δ/δ isomer, we attempted to reduce the acidity of the solution. However, when the pH increased, due to the higher concentration of Cu^2+^ in the solution, it became easier to obtain a precipitate and the expected structure could not be obtained.

### 2.3. Construction of 2D Structures Based on Triol-Ligand Decorated Anderson-Evans POMs

**Figure 3:**
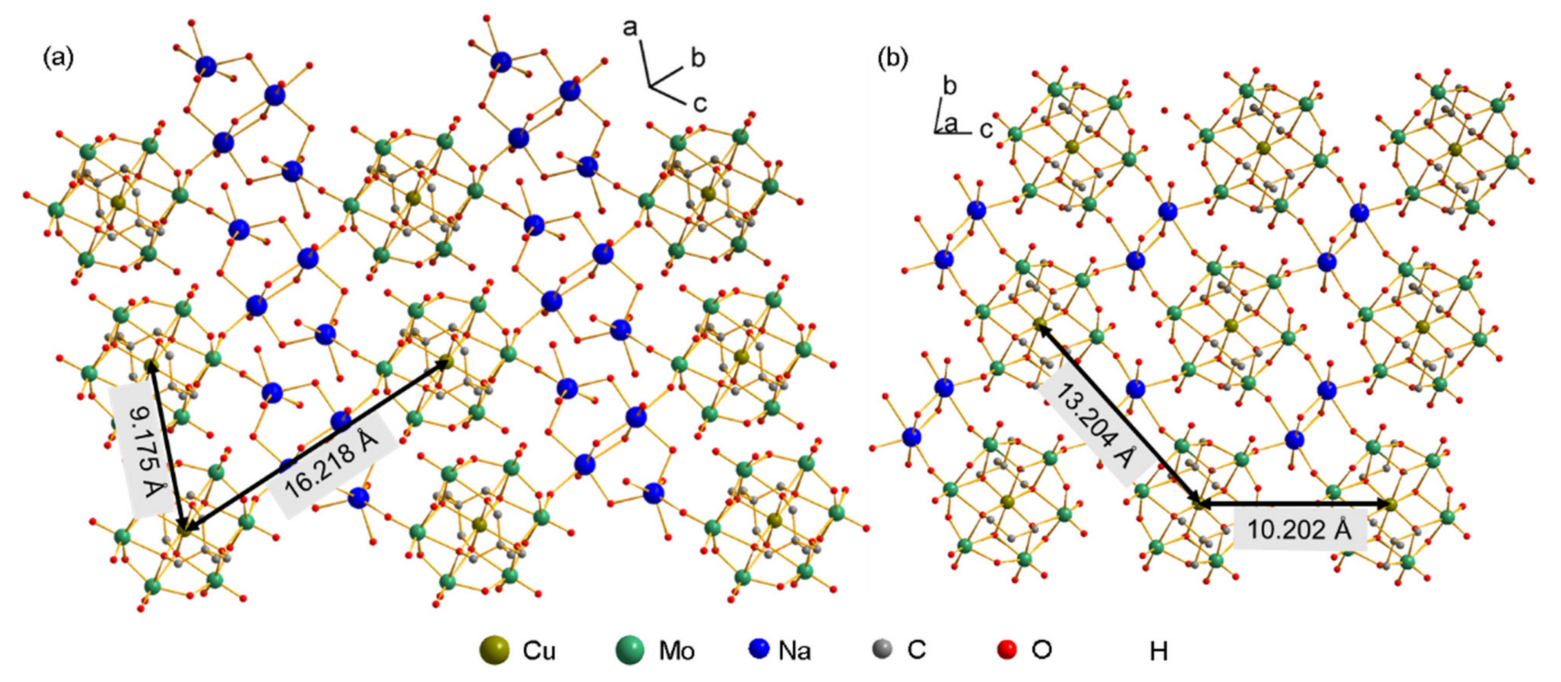
Ball-and-stick representations of compounds (**a**) **8** and (**b**) **9**, showing their 2D planar structures. Numbers in the diagram represent the distances of adjacent clusters in two dimensions. All H atoms except those attaching to tri-bridging O atoms in polyanions are omitted for clarity.

As mentioned above, when Cu^2+^ is used as a linker, its small coordination range represents a disadvantage for the formation of a 2D structure, and its synthetic environment with a low pH value is also not conducive to obtaining the triol ligand modified Anderson-Evans clusters in the δ/δ isomer. Considering these points, we selected Na_4_Mo_8_O_26_ as the Mo source, and used Na^+^ with a high solubility in a wide pH range in an aqueous solution and a large range of connection to obtain 2D structures based on triol-ligand modified Anderson-Evans clusters in δ/δ and χ/χ isomer. As expected, when the pH of the solution was 5, we obtained compound **8**, in which the triol ligand double-sided decorated Anderson-Evans polyanion was an δ/δ isomer. In this case, four Na^+^ ions aggregated to form a {Na_4_(H_2_O)_14_} cluster, which linked the adjacent four polyanions through the terminal O atoms into a 2D planar network along the (01$\bar{1}$) direction (Figure 3a). When the pH of the solution was lowered to 3.5, the triol ligand modified Anderson-Evans polyanions in χ/χ isomer was obtained as in compound **9**. In this adduct, two Na^+^ ions form a dimer {Na_2_(H_2_O)_4_} and connect the adjacent four polyanions to form a 2D planar structure extending along the (100) direction (Figure 3b). It can be seen from the above two examples that the charges of the anions and cations have obvious matching characteristics. For the δ/δ isomer, because the charge of the polyanion was −4, four one-charged Na^+^ ions combined to form a tetramer, thereby matching the charge with the anion. While for the χ/χ isomer, because the charge of polyanion was −2, two Na^+^ ions combined to form a dimer with two positive charges, and were neutralized with the anion in a 1:1 mode. In addition, compared with Cu^2+^, the tetramer and dimer of Na^+^ are larger and have wider connecting ranges, so that four polyanions can be uniformly arranged around them to form 2D planar structures, which can also be seen as the adjacent 1D chains connecting to each other to form 2D structures.

**Figure 4:**
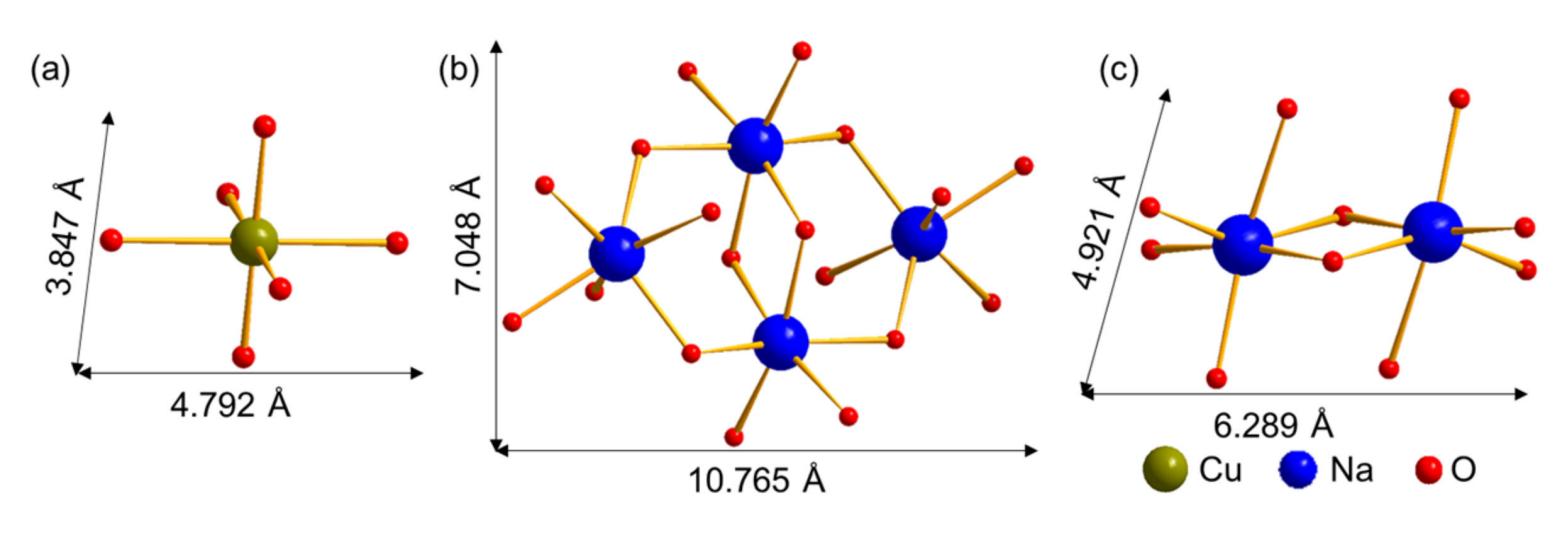
Ball-and-stick representations of coordination environments of linkers for compounds (**a**) **6** and **7**, (**b**) **8**, and (**c**) **9**, showing their different sizes and bridging manners.

The size of the linking group and the bridging manner have an important effect on the distances between the order of the arranged anions. When the linking group was a Cu^2+^ ion, as in compounds **6** and **7**, the distances between two adjacent polyanions in the 1D chains were 14.090(2) Å and 13.312(2) Å, respectively (Figure S16). The slight difference between the two values is due to the steric hindrance caused by the triol ligand coordinated with linking Cu^2+^ (Figure 2). For inter-chains, and the distances between the two adjacent polyanions in compounds **6** and **7** are 11.128(2) Å and 8.872(2) Å, respectively (Figure S16). For compound **7**, the smaller distance between the adjacent polyanions is mainly due to the different orientations of the two polyanions in adjacent chains, which reduces the steric hindrance based on the rotation of one cluster, so that the distance between the two center heteroatoms decreases. When the linking group was a {Na_4_(H_2_O)_14_} tetramer, as in Compound **8**, the closest distances of the two adjacent polyanions in the 2D structure were 16.218(2) Å and 9.175(2) Å, respectively (Figure 3). In compound **9**, when the linking group was a {Na_2_(H_2_O)_4_} dimer, the distances between the two adjacent polyanions were 13.204(2) Å and 10.202(2) Å, respectively. As shown in Figure 4, from the chemical environments of linkers in compounds **6–9**, we can see that when the bridging group is a single metal ion, the mode of bridging polyanions is simple, and the distance between polyanions mainly depends on the radius of the bridging metal ions. When the linker is a cluster formed by multiple metal ions, the bridging range becomes significantly larger, and it can interact with the polyanion through various modes, so that the polyanions have richer assembled structures.

### 2.4. Hirshfled Surface Analysis

As demonstrated by the analysis above, the triol-ligand modified Anderson-Evans POMs can serve as building blocks for the construction of 1D or 2D assemblies based on different metal ions and their various combinations. In fact, with similar building blocks, 3D assemblies have also been prepared, through which ionic frameworks form and exhibit selective adsorption capacity to CO_2_ over N_2_, H_2_ and CH_4_ [39,40]. All these 1D to 3D assemblies are constructed based on a strong ionic bond or coordination bond and provide firm connections between each other, while in the absence of metal ions, there are still relatively weak contacts between building blocks, which also have an important influence on their packing styles. Herein, the Hirshfeld surface analysis was applied to illustrate the supramolecular interactions between triol-ligand modified Anderson-Evans polyanions. To exclude the effects of metal ions, only compounds **1**–**5** were analyzed in which an ammonium ion serves as the counter cation and cannot provide obvious directional interactions to the assembly behavior of building blocks such as that of metal ions. As the important supramolecular interactions, the hydrogen bonds in compounds **1**–**5** are summarized in Tables S1–S5 in the Supplementary Materials.

Hirshfeld surfaces mapped with the *d*_norm_ of compounds **1**–**5** were firstly investigated, in which *d*_norm_ was the normalized sum of *d*_i_ and *d*_e_, and is defined as follows [41]:

*d*_norm_ = (*d*_i_ − *r*_I_^vdw^)*/r*_I_^vdw^ + (*d*_e_ − *r*_E_^vdw^)*/r*_E_^vdw^

**Figure 5:**
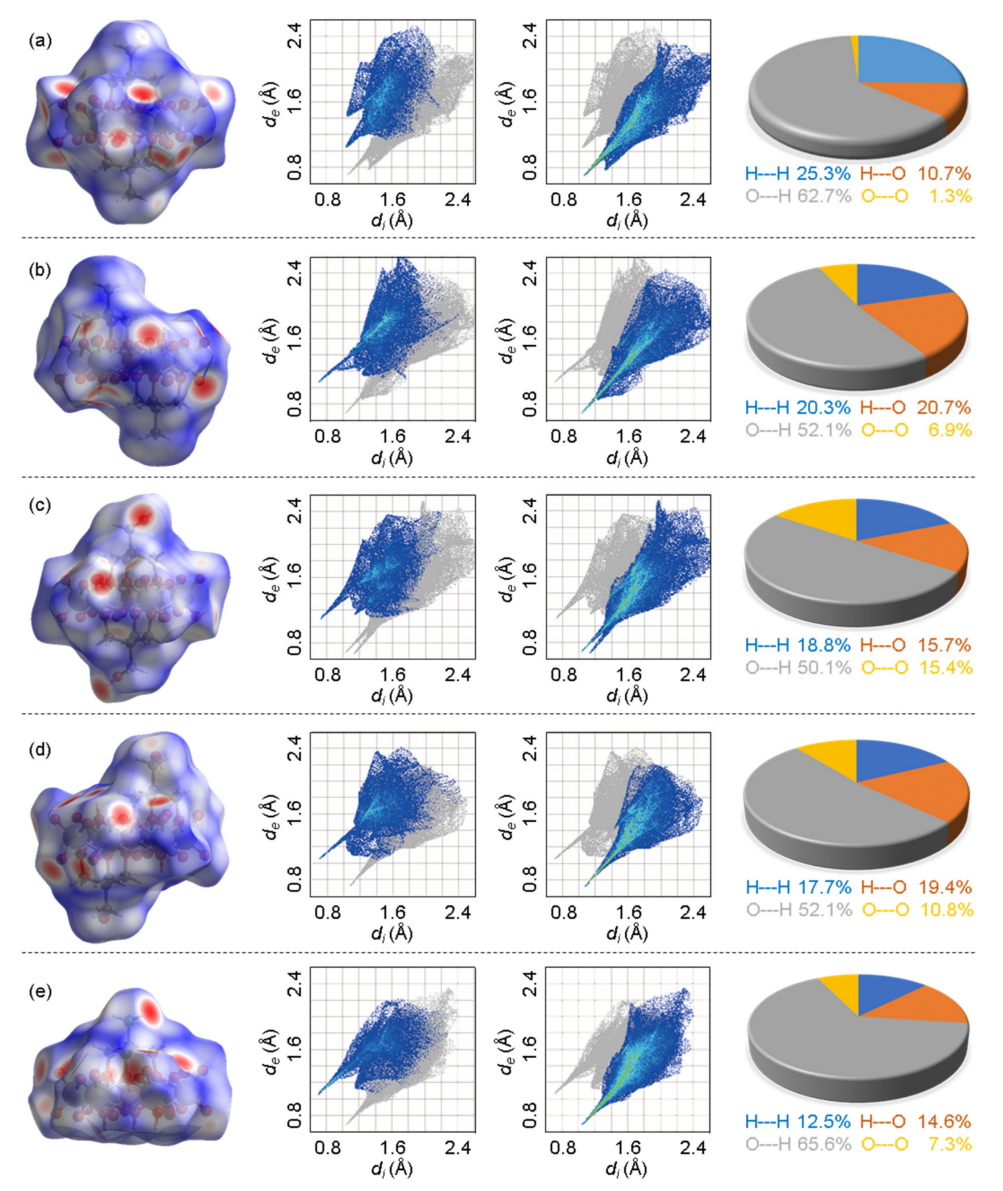
Hirshfeld analysis results for compounds (**a**) **1**, (**b**) **2**, (**c**) **3**, (**d**) **4**, and (**e**) **5**, respectively. For each column, from left to right, the four images are as follows: Hirshfeld surface mapped with *d*_norm_ in a transparent mode, colored 2D fingerprint plot showing contacts between H (internal to the surface) and H and O (external to the surface), colored 2D fingerprint plot showing contacts between O (internal to the surface) and H and O (external to the surface), and percentage distribution of various short contacts (the detailed percentages of contacts are labeled with the corresponding colors).

*d*_i_ is the distance from Hirshfeld surface to the nearest atom I internal to the surface, *d*_e_ is the distance from Hirshfeld surface to the nearest atom E external to the surface, *r*_I_^vdw^ is the van der Waals radius of the nearest atom I closest to and inside the Hirshfeld surface, and *r*_E_^vdw^ is the van der Waals radius of the nearest atom E closest to and outside the Hirshfeld surface. As shown in Figure 5, when a methyl-containing triol ligand was used in compounds **1** and **2**, the main interaction sites (marked with red cones) were concentrated at the lateral edge of the disk-shaped cluster, where the terminal O atoms can serve as hydrogen bonding acceptors, while for compounds with hydroxyl or protonated amino groups such as compounds **3**–**5**, their ability to serve as hydrogen bonding donors resulted in a relatively uniform distribution of strong contact sites surrounding the cluster.

Hirshfeld surface images provide qualitative descriptions of the supramolecular interactions of clusters, and 2D fingerprint plots in the range of 0.6–2.6 Å for *d*_i_ and *d*_e_ were further applied to analyze the detailed contacts in quantitative accuracy (Figure 5). All five compounds have some common characteristics, such as for interactions of H atoms internal to the surface with H or O atoms external to the surface with generally small *d*_i_ values (0.6–2.0 Å) and large *d*_e_ values (1.0–2.4 Å), while O atoms internal to the surface with H or O atoms external to the surface have an opposite trend with large *d*_i_ values (1.0–2.6 Å) and small *d*_e_ values (0.6–2.4 Å). These features indicate that the triol-ligand modified clusters are preferred as hydrogen bonding acceptors than donors, which is also in accordance with the traits of O-rich surfaces. It should be noted that the modification sites of the triol ligand also have an obvious influence on the intermolecular interactions. For example, compounds **1** and **2** have the same triol ligand on the cluster and different positions to the δ/δ and χ,/χ isomers, respectively. In compound **1**, trio ligands were located at the center position of the cluster, which enlarged the distances of methene and methyl H to other species and results in relatively large *d*_i_ and *d*_e_ values (both more than 1.0 Å) in 2D fingerprint plot. As a comparison, in compound **2**, triol ligands were located at the edge of the disk-like cluster, which enhanced their abilities as hydrogen-bonding donors and generated relatively stronger contacts between H atoms internal to the surface with H and O atoms external to the surface. In addition, the χ,/χ isomer in compound **2** also provides two extra protonated μ_3_-O atoms as hydrogen bonding donors compared with compound **1**, which are important components of short contacts. On the contrary, the positions of triol ligands have little influence on the hydrogen-bonding-acceptor ability of the cluster, and the 2D fingerprint plots showing contacts between O (internal to the surface) and H and O (external to the surface) of compounds **1** and **2** are very similar. Lastly, the discoid distributions of various contacts indicate that all five triol-ligand modified clusters are mainly used as hydrogen-bonding acceptors with the percentages of O---H contacts ranging from 50.1% to 65.6%.

## 3. Materials and Methods

### 3.1. General Methods and Materials

All chemicals were purchased from Aladdin and used without further purification. Double distilled water was used in all the reactions. Fourier transform infrared spectra were obtained with a Bruker Vertex 80v spectrometer equipped with a DTGS detector with a resolution of 4 cm^−1^ in KBr pellets. Elemental analysis of C, H, and N was conducted using a vario MICRO cube from Elementar Company of Germany. Elemental analysis for Cu, Mo and Na was carried out on a PLASMA-SPEC (I) inductively coupled plasma atomic emission spectrometer. Thermogravimetric analysis curves were obtained with a Q500 Thermal Analyzer (New Castle TA Instruments, New Castle, DE, USA) in a flowing N_2_ under a heating rate of 10 °C∙min^−1^. Powder X-ray diffraction data were recorded on a Rigaku SmartLab X-ray diffractometer using Cu K_α_ radiation at a wavelength of 1.54 Å. Single-crystal X-ray diffraction data were collected on a Bruker D8 VENTURE diffractometer with graphite-monochromated Mo K_α_ (λ = 0.71073 Å) at 293 K. All crystals were solved by *SHELXT* and refined by full-matrix-least-squares fitting for *F*^2^ using the Olex2 software [42,43]. All non-H atoms were refined with anisotropic thermal parameters. A summary of the crystallographic data and structural refinements for compounds **1**–**9** is listed in Table 1. The detailed CCDC 2162383–2162391 contains the supplementary crystallographic data for this paper. These data can be obtained free of charge from www.ccdc.cam.ac.uk/data_request/cif, accessed on 27 March 2022, or by emailing data_request@ccdc.cam.ac.uk, or by contacting The Cambridge Crystallographic Data Centre, 12 Union Road, Cambridge CB2 1EZ, UK.

### 3.2. Synthesis of Compounds

#### 3.2.1. Synthesis of [NH_4_]_4_[CuMo_6_O_18_(CH_3_C(CH_2_O)_3_)_2_]∙5.5H_2_O (**1**)

A mixture of CuCl_2_·2H_2_O (0.34 g, 2.00 mmol), (NH_4_)_6_Mo_7_O_24_·4H_2_O (2.47 g, 2.00 mmol) and 0.48 g of CH_3_C(CH_2_OH)_3_ (0.48 g, 4.00 mmol) was dissolved in 20 mL of deionized water under stirring. The pH of the mixture was adjusted to 3~4 with the concentrated HCl, and the resulting solution was heated to 80 °C, and maintained for 2 h. After a hot filtration process, a blue solution was obtained, which provided blue crystals suitable for X-ray single-crystal diffraction analysis after about one day at room temperature. Elemental analysis calcd. (%) for [NH_4_]_4_[CuMo_6_O_18_(CH_3_C(CH_2_O)_3_)_2_]∙5.5H_2_O (Mw = 1332.68 g mol^−1^): C 9.01%, H 3.40%, N 4.20%, Cu 4.77%, and Mo 43.19%; found C 9.20%, H 3.40%, N 4.25%, Cu 4.83%, and Mo 43.86%.

#### 3.2.2. Synthesis of [NH_4_]_2_{CuMo_6_O_16_(OH)_2_[CH_3_C(CH_2_O)_3_]_2_}·2H_2_O (**2**)

The synthetic procedure of compound **2** was similar to that of compound **1** except that the pH of the solution was adjusted to 2. Elemental analysis calcd. (%) for [NH_4_]_2_{CuMo_6_O_16_(OH)_2_[CH_3_C(CH_2_O)_3_]_2_}·2H_2_O (Mw = 1235.58 g mol^−1^): C 9.72%, H 2.61%, N 2.27%, Cu 5.14%, and Mo 46.59%; found C 9.31%, H 2.80%, N 2.31%, Cu 5.21%, and Mo 46.76%.

#### 3.2.3. Synthesis of [NH_4_]_4_[CuMo_6_O_18_(HOCH_2_C(CH_2_O)_3_)_2_]∙5.5H_2_O (**3**)

The synthetic procedure of compound **3** was similar with that of compound **1** except that HOCH_2_C(CH_2_OH)_3_ (0.54 g, 4.00 mmol) was used for substituting CH_3_C(CH_2_OH)_3_. Elemental analysis calcd. (%) for [NH_4_]_4_[CuMo_6_O_18_(HOCH_2_C(CH_2_O)_3_)_2_]∙5.5H_2_O (Mw = 1364.68 g mol^−1^): C 8.80%, H 3.32%,N 4.11%, Cu 4.66%, and Mo 42.18%; found C 8.91%, H 3.36%, N 4.18%, Cu 4.70%, and Mo 42.24%.

#### 3.2.4. Synthesis of [NH_4_]_2_{CuMo_6_O_16_(OH)_2_[HOCH_2_C(CH_2_O)_3_]_2_}·4H_2_O (**4**)

The synthetic procedure of compound **4** was similar with that of compound **3** except that the pH of the solution was adjusted to 2. Elemental analysis calcd. (%) for [NH_4_]_2_{CuMo_6_O_16_(OH)_2_[HOCH_2_C(CH_2_O)_3_]_2_}·4H_2_O (Mw = 1303.59 g mol^−1^): C 9.21%, H 2.78%, N 2.15%, Cu 4.87%, and Mo 44.16%; found C 9.28%, H 2.87%, N 2.22%, Cu 4.90%, and Mo 44.21%.

#### 3.2.5. Synthesis of (NH_4_)_3_{CuMo_6_O_18_(OH)_3_[NH_3_C(CH_2_O)_3_]}·6H_2_O (**5**)

CuCl_2_·2H_2_O (0.34 g, 2.00 mmol), (NH_4_)_6_Mo_7_O_24_·4H_2_O (2.47 g, 2.00 mmol) and NH_2_C(CH_2_OH)_3_ (0.48 g, 4.00 mmol) were dissolved in 20 mL of deionized water under stirring. The pH of the mixture was adjusted to about 1.5~2.5 by the concentrated HCl, and the resulting solution was heated to 80 °C gradually for 2 h. A blue solution was obtained with a hot filtration process, and provided blue crystals in one day. Elemental analysis calcd. (%) for [NH_4_]_3_[CuMo_6_O_18_(OH)_3_(NH_3_C(CH_2_O)_3_)_2_]∙6H_2_O (Mw = 1259.55 g mol^−1^): C 3.81%, H 2.88%, N 4.45%, Cu 5.05%, and Mo 45.70%; found C 3.88%, H 2.95%, N 4.55%, Cu 5.10%, and Mo 45.77%.

#### 3.2.6. Synthesis of Cu[CH_3_C(CH_2_OH)_3_]_2_{CuMo_6_O_16_(OH)_2_[CH_3_C(CH_2_O)_3_]_2_}·6H_2_O (**6**)

To a stirred solution of CuCl_2_·2H_2_O (0.34 g, 2.00 mmol) and CH_3_C(CH_2_OH)_3_ (0.48 g, 4.00 mmol) in water (20 mL), MoO_3_ (0.86 g, 12.00 mmol) was added. The pH of the resulting solution was adjusted to 3~4 by the concentrated HCl. After heating the mixture to 80 °C for 2 h, a blue solution was obtained using hot filtration, which generates blue crystals in several days. Elemental analysis calcd. (%) for Cu[CH_3_C(CH_2_OH)_3_]_2_{CuMo_6_O_16_(OH)_2_[CH_3_C(CH_2_O)_3_]_2_}·6H_2_O (Mw = 1575.36 g mol^−1^): C 15.25%, H 3.58%, Cu 8.07%, and Mo 36.54%; found C 15.58%, H 3.65%, Cu 8.14%, and Mo 36.55%.

#### 3.2.7. Synthesis of Cu(H_2_O)_4_{CuMo_6_O_16_(OH)_2_[CH_3_C(CH_2_O)_3_]_2_}·10H_2_O (**7**)

The synthetic procedure of compound **7** was similar with that of compound **6** except that the pH of the solution was adjusted to 2~2.5. Elemental analysis calcd. (%) for Cu(H_2_O)_4_{CuMo_6_O_16_(OH)_2_[CH_3_C(CH_2_O)_3_]_2_}·10H_2_O (Mw = 1479.20 g mol^−1^): C 8.12%, H 3.27%, Cu 8.59%, and Mo 38.92%; found C 8.21%, H 3.35%, Cu 8.64%, and Mo 39.01%.

#### 3.2.8. Synthesis of Na_4_(H_2_O)_14_{CuMo_6_O_18_[CH_3_C(CH_2_O)_3_]_2_}·2H_2_O (**8**)

CuCl_2_·2H_2_O (0.34 g, 2.00 mmol), Na_4_Mo_8_O_26_ (1.92 g, 1.50 mmol) and CH_3_C(CH_2_OH)_3_ (0.48 g, 4.00 mmol) were dissolved in 20 mL of deionized water under stirring. Concentrated HCl was used to adjust the pH of the mixture to 5, and then the resulting solution was heated to 80 °C for 2 h. A blue solution was obtained using a hot filtration process, and generated blue crystals in several days. Elemental analysis calcd. (%) for Na_4_(H_2_O)_14_{CuMo_6_O_18_[CH_3_C(CH_2_O)_3_]_2_}·2H_2_O (Mw = 1541.64 g mol^−1^): C 7.79%, H 3.27%, Na 5.97%, Cu 4.12%, and Mo 37.34%; found C 7.84%, H 3.35%, Na 6.05%, Cu 4.06%, and Mo 37.18%.

#### 3.2.9. Synthesis of Na_2_(H_2_O)_4_{CuMo_6_O_16_(OH)_2_[CH_3_C(CH_2_O)_3_]_2_}·6H_2_O (**9**)

The synthetic procedure of compound **9** was similar with that of compound **8** except that the pH of the solution was adjusted to 3.5. Elemental analysis calcd. (%) for Na_2_(H_2_O)_4_{CuMo_6_O_16_(OH)_2_[CH_3_C(CH_2_O)_3_]_2_}·6H_2_O (Mw = 1389.58 g mol^−1^): C 8.64%, H 2.90%, Na 3.31%, Cu 4.57%, and Mo 41.43%; found C 8.70%, H 2.94%, Na 3.38%, Cu 4.66%, and Mo 41.52%.

## 4. Conclusions

By synthesizing a series of Cu^II^-centered Anderson-Evans POMs modified with triol ligands in the presence of different cations in aqueous solution, ordered assemblies of polyanions from 0D to 2D under cation regulation were successfully realized. The results show that NH_4_^+^ directs the formation of 0D building blocks, and Cu^2+^ leads the 1D chain structures, while Na^+^ dominates 2D networks. During the assembled process, the charge ratio between cations and anions has an important effect on the assembled structures. When the size of a cation is large, its wider connection range can link the four adjacent polyanions together to form a 2D structure, while when the size of cation is small, the polyanions can only be arranged in a 1D chain. The research work in this section not only provides an important method and idea for the construction of an ordered assembly of polyanions, but also lays the foundation for the development of specific functional systems.

## Figures and Tables

**Table 1:** The summary of crystal data and structure refinements for compounds **1**–**9**.

	1	2	3	4	5
Formula	[NH_4_]_4_[CuMo_6_O_18_(CH_3_C(CH_2_O)_3_)_2_]∙5.5H_2_O	[NH_4_]_2_{CuMo_6_O_16_(OH)_2_[CH_3_C(CH_2_O)_3_]_2_}·2H_2_O	[NH_4_]_4_[CuMo_6_O_18_(HOCH_2_C(CH_2_O)_3_)_2_]∙5.5H_2_O	[NH_4_]_2_{CuMo_6_O_16_(OH)_2_[HOCH_2_C(CH_2_O)_3_]_2_}·4H_2_O	(NH_4_)_3_{CuMo_6_O_18_(OH)_3_[NH_3_C(CH_2_O)_3_]}·6H_2_O
F. W.	1332.68	1235.55	1364.68	1303.59	1259.55
S. G.	*P*4_2_/*n*	*P*2_1_/*n*	*P*4_2_/*n*	*P* $\bar{1}$	*P* $\bar{1}$
a (Å)	16.6461(3)	8.8076(5)	16.6651(3)	8.7546(2)	10.9061(8)
b (Å)	16.6461(3)	17.1452(9)	16.6651(3)	9.8069(3)	12.1946(9)
c (Å)	12.9739(4)	11.1324(6)	13.0675(5)	10.3904(3)	12.9648(9)
α (deg)	90	90	90	95.823(1)	72.139(3)
β (deg)	90	110.302(2)	90	98.085(1)	62.020(2)
γ (deg)	90	90	90	112.510(1)	82.620(3)
V (Å^3^)	3595.0(2)	1544.0(2)	3629.2(2)	804.1(1)	1510.8(2)
Z	4	2	4	1	2
D_c_ (g cm^−3^)	2.462	2.658	2.498	2.692	2.769
*F*(000)	2600	1190	2664	631	1218
Reflections coll./unique	43,727/4424	6373/1635	27,878/3123	25,824/3694	46,661/7481
*R*_int_	0.0172	0.0255	0.0291	0.0224	0.0275
GOF on *F^2^*	1.146	1.251	1.310	1.079	1.045
*R*_1_^a^ [*I* > 2*σ*(*I*)]	0.0176	0.0483	0.0470	0.0197	0.0261
*wR*_2_^b^ (all data)	0.0438	0.1082	0.1063	0.0517	0.0779
CCDC no.	2,162,383	2,162,384	2,162,385	2,162,386	2,162,387
	6	7	8	9	
Formula	Cu[CH_3_C(CH_2_OH)_3_]_2_{CuMo_6_O_16_(OH)_2_[CH_3_C(CH_2_O)_3_]_2_}·6H_2_O	Cu(H_2_O)_4_{CuMo_6_O_16_(OH)_2_[CH_3_C(CH_2_O)_3_]_2_}·10H_2_O	Na_4_(H_2_O)_14_{CuMo_6_O_18_[CH_3_C(CH_2_O)_3_]_2_}·2H_2_O	Na_2_(H_2_O)_4_{CuMo_6_O_16_(OH)_2_[CH_3_C(CH_2_O)_3_]_2_}·6H_2_O	
F. W.	1575.36	1479.20	1541.64	1389.58	
S. G.	*P* $\bar{1}$	*P*2_1_/*c*	*P* $\bar{1}$	*P* $\bar{1}$	
a (Å)	10.4512(9)	13.3123(9)	9.1747(2)	9.6583(3)	
b (Å)	10.7305(9)	13.4631(8)	10.4014(2)	10.2021(4)	
c (Å)	11.1280(9)	11.5585(8)	11.6954(2)	10.5633(4)	
α (deg)	110.643(3)	90	96.479(1)	78.845(1)	
β (deg)	91.194(4)	99.210(3)	97.716(1)	72.565(1)	
γ (deg)	105.688(3)	90	92.964(1)	67.093(1)	
V (Å^3^)	1115.0(2))	2044.9(2)	1096.4(1)	911.4(1)	
Z	1	2	1	1	
D_c_ (g cm^−3^)	2.672	2.402	2.335	2.532	
*F*(000)	774	1444	755	675	
Reflections coll./unique	16,695/5112	32,538/5059	14,364/5416	114,40/4184	
*R*_int_	0.0219	0.0296	0.0205	0.0212	
GOF on *F^2^*	1.032	1.032	1.039	1.065	
*R*_1_^a^ [*I* > 2*σ*(*I*)]	0.0205	0.0190	0.0219	0.0222	
*wR*_2_^b^(all data)	0.0528	0.0481	0.0544	0.0552	
CCDC no.	216,238	2,162,389	2,162,390	2,162,391	

^a^*R*_1_ = Σ||*F*_o_| − |*F*_c_||/Σ|*F*_o_|. ^b^ *wR*_2_ = Σ[*w*(*F*_o_^2^ − *F*_c_^2^)^2^]/Σ[*w*(*F*_o_^2^)^2^]^1/2^.

## Data Availability

Crystallographic data can be obtained free of charge from the Cambridge Crystallographic Data Centre via www.ccdc.cam.ac.uk/data_request/cif, accessed on 27 March 2022.

## Supplementary Materials

The following supporting information is available online at https://www.mdpi.com/article/10.3390/molecules27092933/s1. Figure S1: The schematic drawing of possible decoration types when triol ligands bind to an Anderson-Evans POM cluster, where the blue octahedron represents {MO_6_} (M = Mo or W) and yellow octahedron denotes heteroatom-oxygen {XO_6_}; Figure S2: FT-IR spectra of compounds **1**–**9**; Figures S3–S11: TGA curves of compounds **1**–**9**; Figures S12–S14: XRD patterns of as-synthesized compounds **1**–**9** and their simulated patterns from the corresponding single-crystal X-ray diffraction data; Figure S15: Two possible arrangements of linkers and building blocks with 1:1 charge ratio. Spheres and disks represent linkers and building blocks, respectively. The solid and dotted lines show the interactions between the adjacent components; Figure S16: Ball-and-stick representations of compounds (a) **6** and (b) **7**, showing their neighboring chains. Numbers in the diagram represent the distances of adjacent clusters. All H atoms except those attached to tri-bridging O atoms in polyanions are omitted for clarity. Tables S1–S5: Hydrogen bonds for compounds **1**–**5**.

## Sample Availability

Samples of the compounds are available from the authors.
