# Supplementary material for: Cations Modulated Assembly of Triol-Ligand Modified Cu-Centered Anderson-Evans Polyanions
Source: Molecules. 2022 May 4;27(9):2933. doi: 10.3390/molecules27092933 (PMC9101508; doi:10.3390/molecules27092933)
Supplement: Supplementary file 1 [file molecules-27-02933-s001.zip › molecules-1707892-supplementary.pdf]

Supporting Information

Cations Modulated Assembly of Triol−Ligand Modified Cu−Centered Anderson−Evans Polyanions

Yiran Wang, Fengxue Duan, Xiaoting Liu and Bao Li*

State Key Laboratory of Supramolecular Structure and Materials, College of Chemistry, Jilin University, Changchun 130012, P. R. China

E−mail: libao@jlu.edu.cn

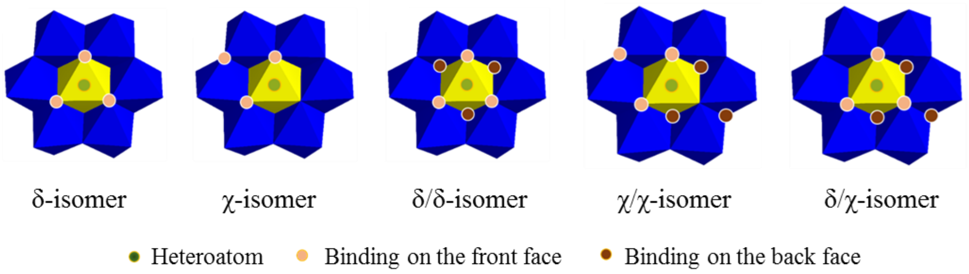

Figure S1. The schematic drawing of possible decoration types when triol ligands bind to an Anderson−Evans POM cluster, where the blue octahedron represents {MO_6} (M = Mo or W) and yellow octahedron denotes heteroatom−oxygen {XO_6} [1].

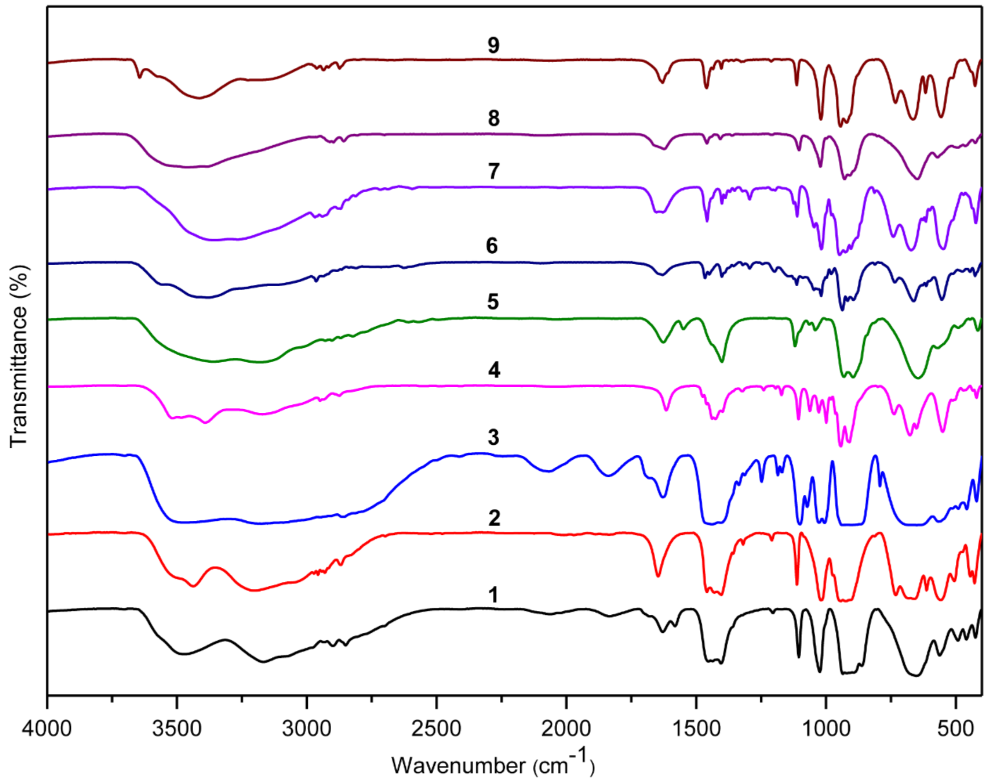

Figure S2. FT−IR spectra of compounds **1**−**9**.

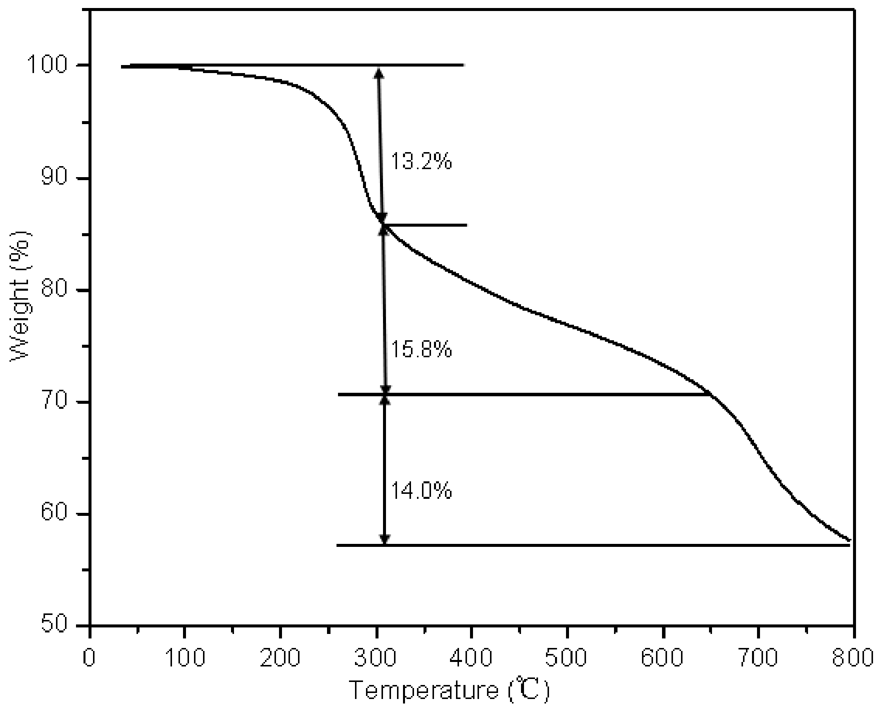

Figure S3. TGA cure of compound **1**.

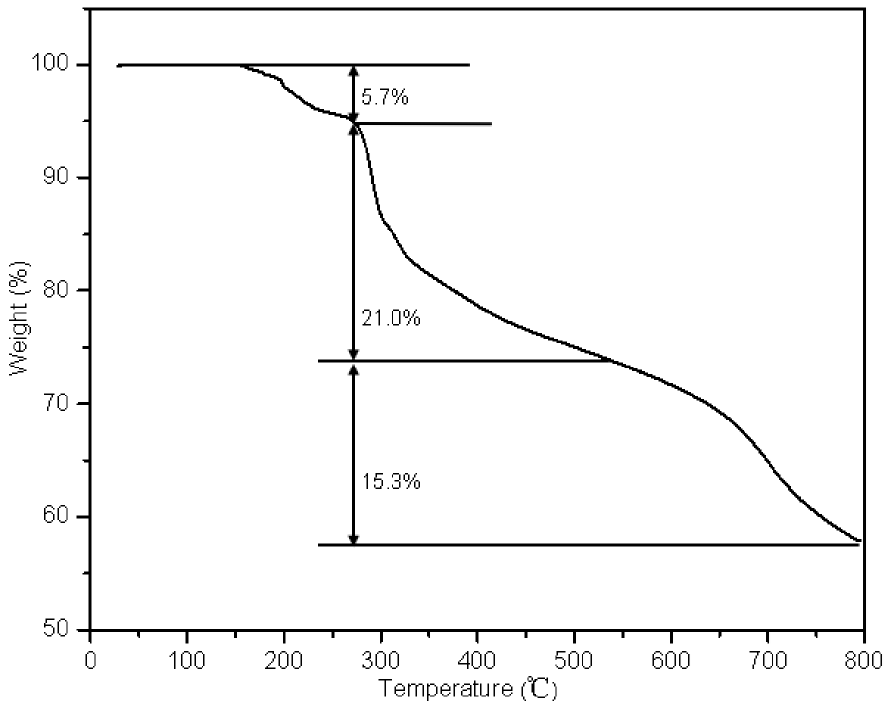

Figure S4. TGA cure of compound **2**.

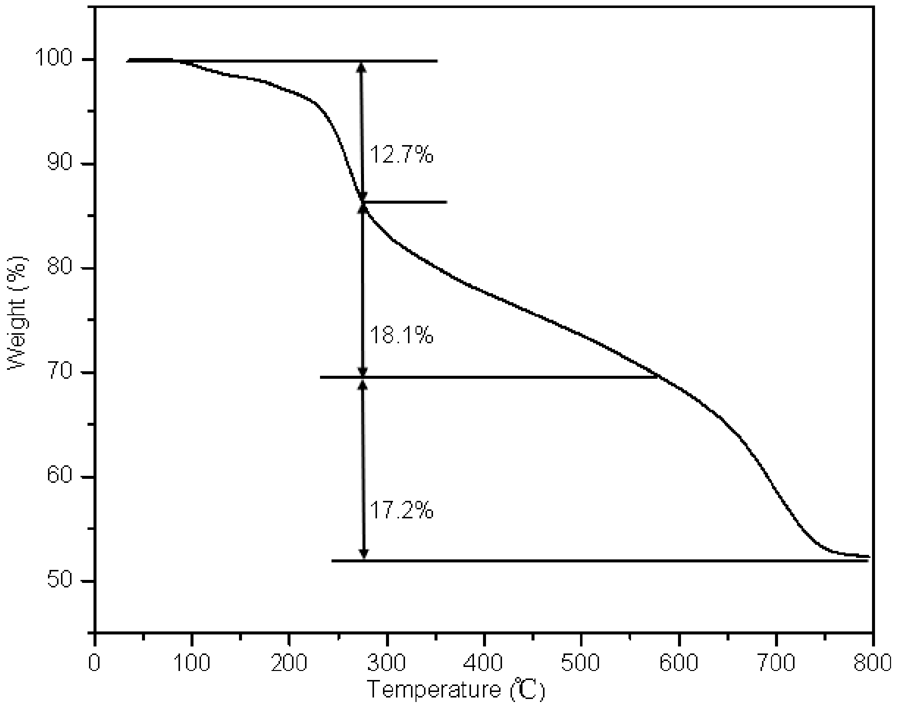

Figure S5. TGA cure of compound **3**.

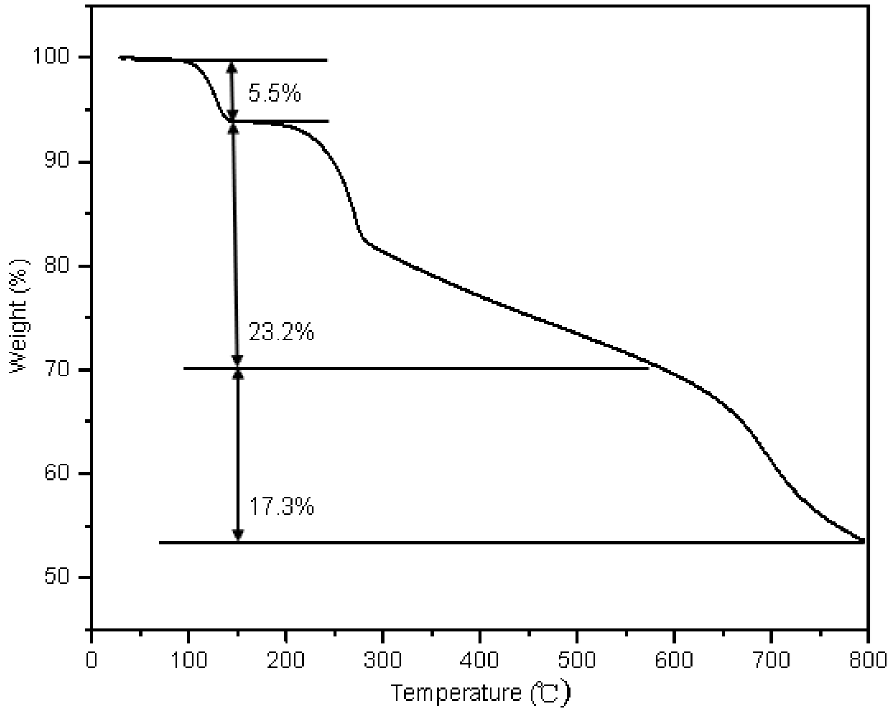

Figure S6. TGA cure of compound **4**.

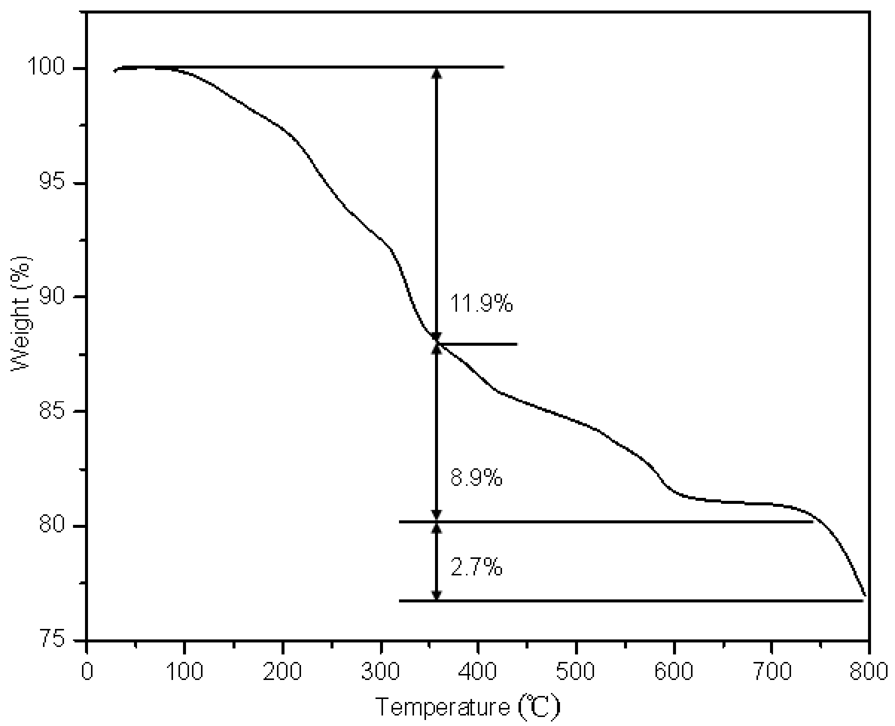

Figure S7. TGA cure of compound **5**.

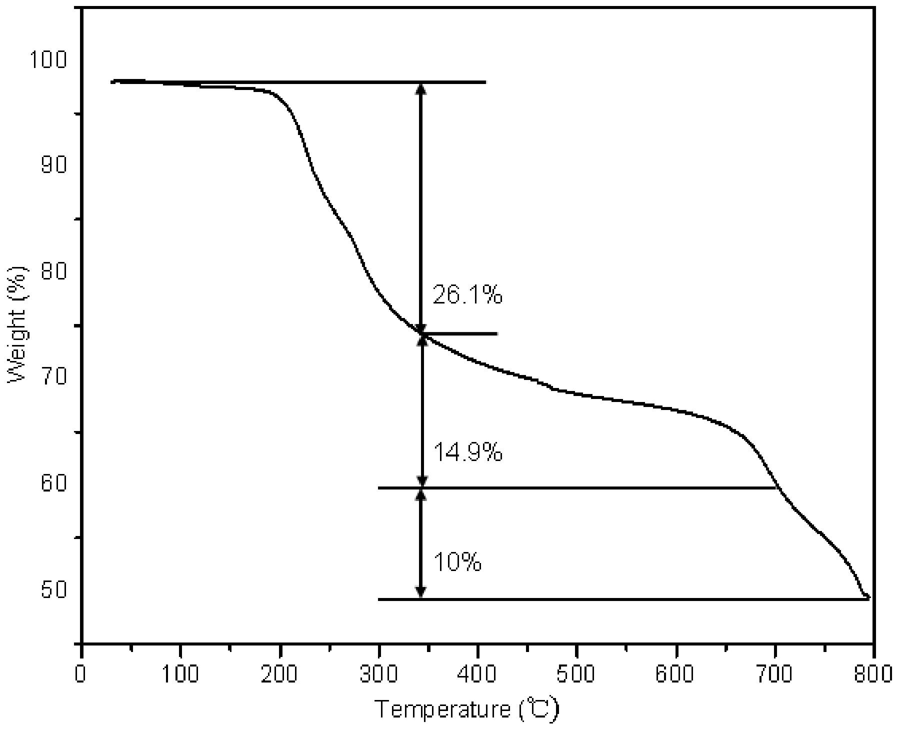

Figure S8. TGA cure of compound **6.**

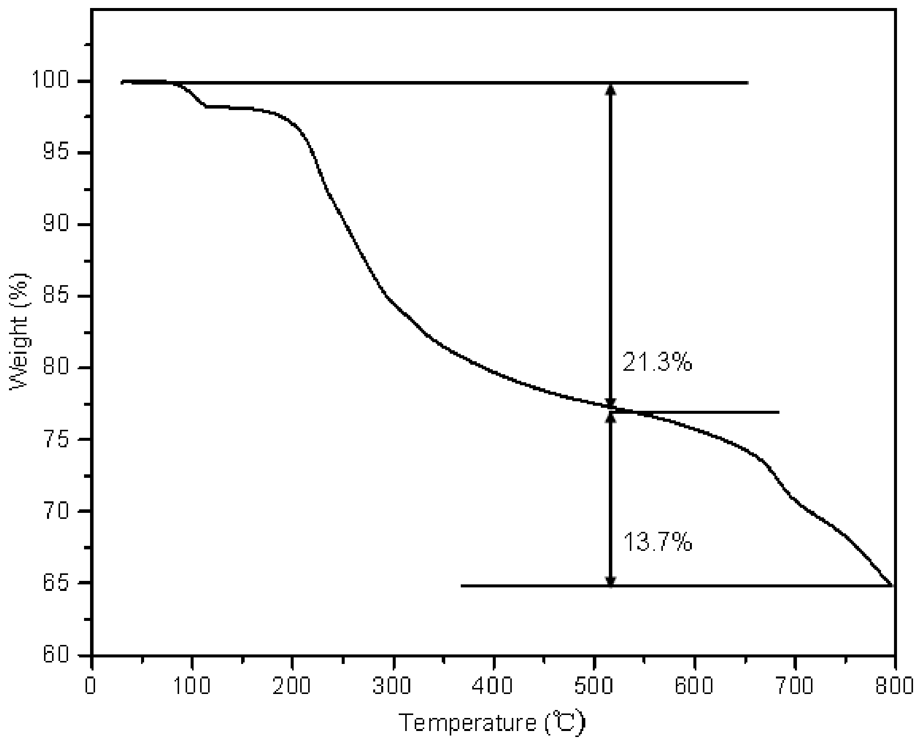

Figure S9. TGA cure of compound **7**.

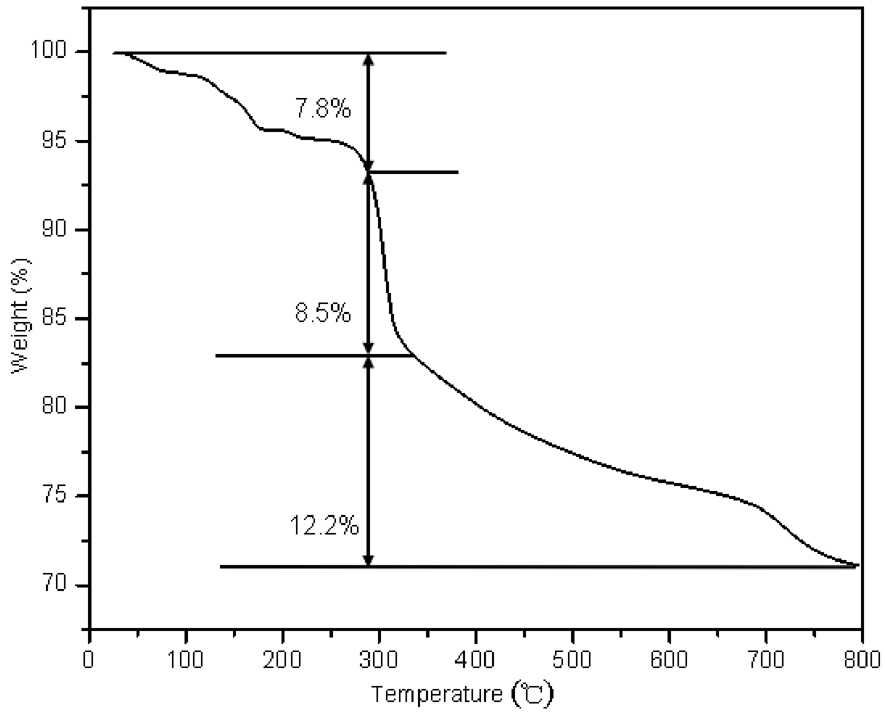

Figure S10. TGA cure of compound **8**

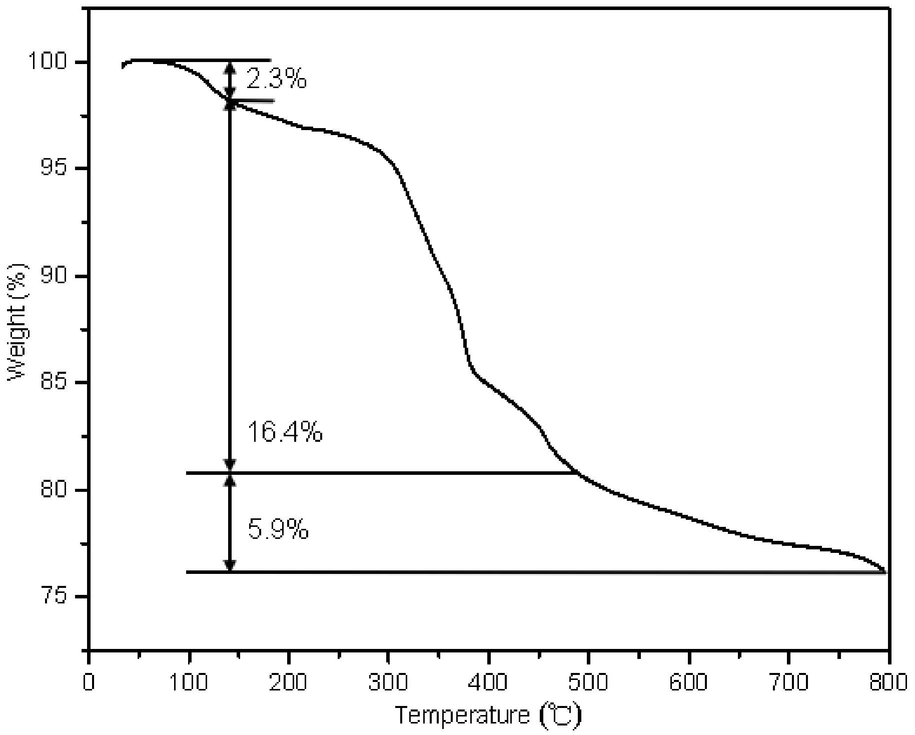

Figure S11. TGA cure of compound **9**

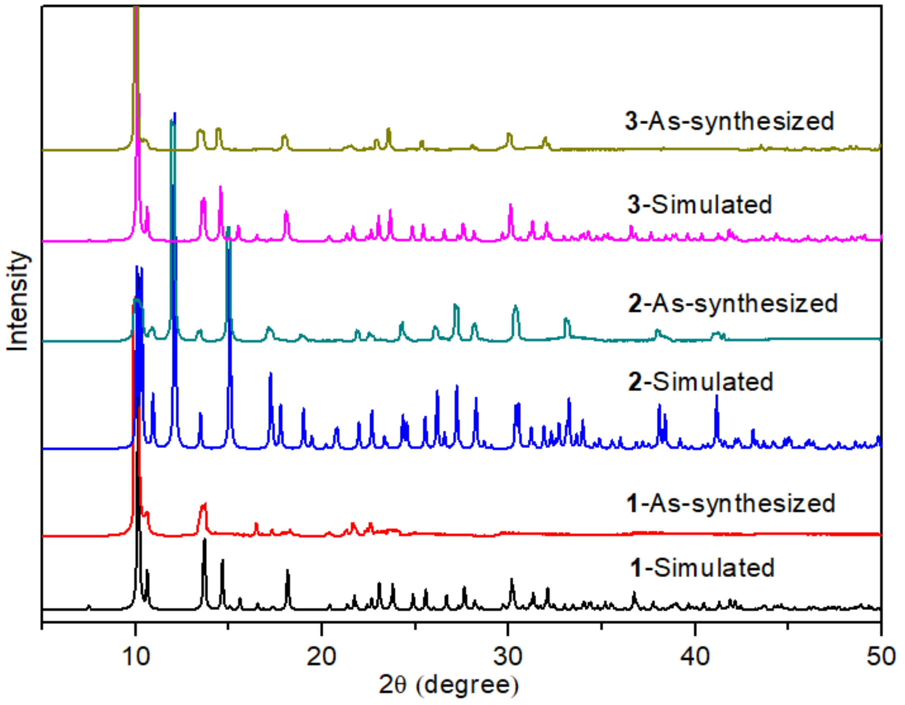

Figure S12. PXRD patterns of as−synthesized compounds **1**−**3** and their simulated patterns from the corresponding single−crystal X−ray diffraction data.

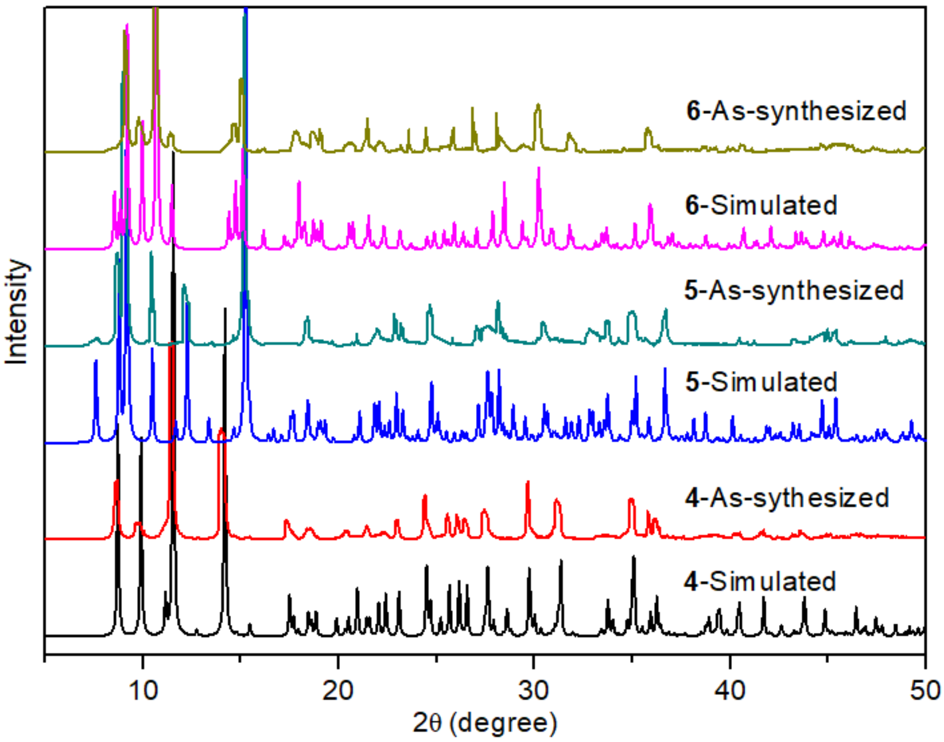

Figure S13. PXRD patterns of as−synthesized compounds **4−6** and their simulated patterns from the corresponding single−crystal X−ray diffraction data.

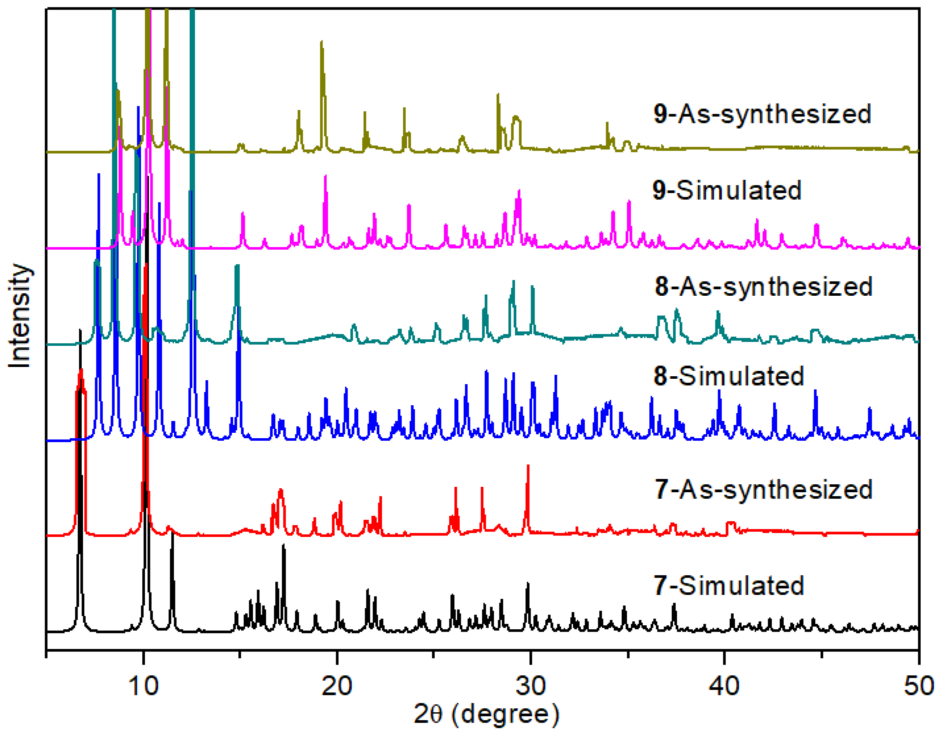

Figure S14. PXRD patterns of as−synthesized compounds **7−9** and their simulated patterns from the corresponding single−crystal X−ray diffraction data.

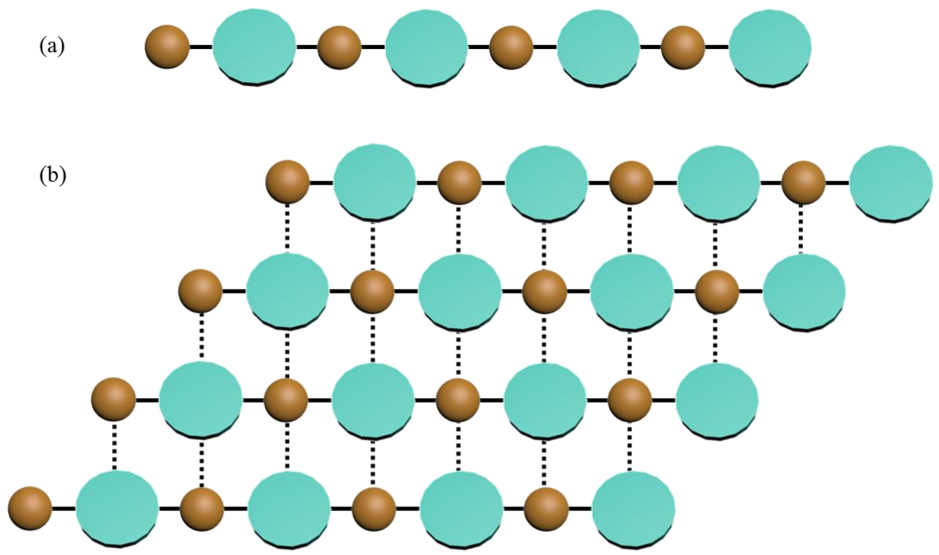

Figure S15. Two possible arrangements of linkers and building blocks with 1:1 charge ratio. Spheres and disks represent linkers and building blocks, respectively. The solid and dotted lines show the interactions between the adjacent components.

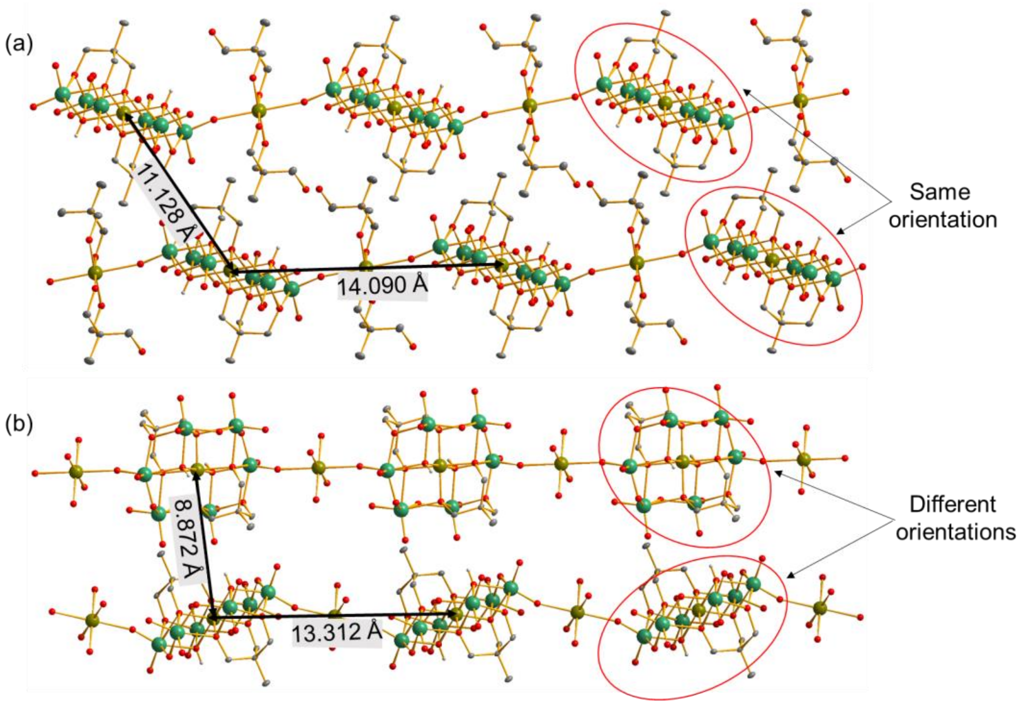

Figure S16. Ball−and−stick representations of compounds (a) **6** and (b) **7**, showing their neighboring chains. Numbers in the diagram represent the distances of adjacent clusters. All H atoms except those attaching to tri−bridging O atoms in polyanions are omitted for clarity.

Table S1. Hydrogen bonds for compound **1** [Å and °].

D−H⋯A	d(D−H)	d(H⋯A)	d(D⋯A)	<(DHA)
N1−H1C⋯O14	0.89	2.02	2.816(4)	148.3
N1−H1D⋯O9#1	0.89	1.98	2.864(3)	172.5
N1−H1E⋯O7#2	0.89	2.00	2.877(3)	167.8
N1−H1F⋯O15	0.89	1.94	2.828(4)	179.7
N2−H2D⋯O16	0.89	2.01	2.783(4)	144.6
N2−H2E⋯O2#3	0.89	2.01	2.874(3)	163.1
N2−H2F⋯O4	0.89	1.89	2.743(2)	161.4
O13−H13A⋯O6#4	0.85	2.18	2.810(2)	131.3
O14−H14B⋯N1#5	0.85	2.05	2.816(4)	150.1
O15−H15A⋯O8	0.85	1.91	2.755(3)	170.2
O16−H16A⋯O7	0.85	2.14	2.877(3)	145.6
O16−H16B⋯O15#4	0.85	2.03	2.844(4)	159.6

Symmetry transformations used to generate equivalent atoms:

#1 y+1/2,−x+1,z+1/2 #2 −y+1,x−1/2,z+1/2 #3 −y+3/2,x,−z−1/2 #4 −y+3/2,x,−z+1/2

#5 −x+3/2,−y+1/2,z

Table S2. Hydrogen bonds for compound **2** [Å and °].

D−H⋯A	d(D−H)	d(H⋯A)	d(D⋯A)	<(DHA)
N1−H1C⋯O9#1	0.89	2.17	2.805(1)	128.2
N1−H1D⋯O8#2	0.89	2.21	2.862(1)	129.8
N1−H1E⋯O2#3	0.89	1.85	2.719(1)	165.2
N1−H1F⋯O4	0.89	1.84	2.733(1)	179.7
O6−H6⋯O13#2	0.85	1.91	2.763(1)	179.3
O13−H13B⋯O12#4	0.85	2.04	2.867(1)	163.8

Symmetry transformations used to generate equivalent atoms:

#1 x+1,y,z #2 −x+1,−y+2,−z+1 #3 x+1/2,−y+3/2,z+1/2 #4 −x,−y+2,−z+1

Table S3. Hydrogen bonds for compound **3** [Å and °].

D−H···A	d(D−H)	d(H···A)	d(D···A)	<(DHA)
N1−H1C···O16	0.89	2.13	2.779(2)	128.8
N1−H1F···O5	0.89	1.84	2.728(9)	179.9
N2−H2D···O15	0.89	2.22	2.793(1)	121.4
O13−H13···O8#1	0.82	1.99	2.801(9)	168.0
O15−H15A···O13	0.85	1.93	2.720(8)	153.4
O15−H15B···N2	0.85	1.98	2.793(1)	160.2
O16−H16B···O17	0.85	2.25	2.800(2)	123.5

Symmetry transformation used to generate equivalent atom:

#1 y−1/2,−x+1,z+1/2

Table S4. Hydrogen bonds for compound **4** [Å and °].

D−H A	d(D−H)	d(H···A)	d(D···A)	<(DHA)
C1−H1B···O11#1	0.97	2.47	2.954(3)	110.8
N1−H1C···O1#2	0.89	2.01	2.840(4)	155.5
N1−H1D···N1#3	0.89	2.67	3.222(5)	121.2
N1−H1D···O7#4	0.89	2.13	2.878(5)	141.2
N1−H1E···O10#5	0.89	2.32	3.034(4)	137.0
N1−H1E···O13#6	0.89	2.42	3.113(3)	135.0
N1−H1F···O14	0.89	1.90	2.790(3)	179.6
O12−H12···O15#1	0.93	1.82	2.731(4)	163.5
O13−H13···N1#7	0.82	2.68	3.274(5)	130.8
O14−H14A···O11#8	0.85	2.03	2.876(5)	173.5
O14−H14B···O2	0.85	1.94	2.780(4)	167.3
O15−H15A···O14	0.85	2.08	2.876(5)	156.3
O15−H15B···O8#8	0.85	2.02	2.779(4)	148.0

Symmetry transformations used to generate equivalent atoms:

#1 −x+1,−y+1,−z+1 #2 x,y−1,z #3 −x+2,−y,−z+2 #4 −x+2,−y+1,−z+2

#5 x,y−1,z+1 #6 −x+1,−y+1,−z+2 #7 x,y+1,z #8 −x+2,−y+1,−z+1

Table S5. Hydrogen bonds for compound **5** [Å and °].

D−H···A	d(D−H)	d(H···A)	d(D···A)	<(DHA)
N1−H1D···O21#1	0.89	1.90	2.784(4)	172.5
N1−H1C···O27#2	0.89	1.99	2.815(5)	154.3
N1−H1E···O29#3	0.89	1.87	2.757(4)	176.0
N2−H2F···O6	0.89	1.92	2.808(3)	179.9
N2−H2E···O25	0.89	1.95	2.804(5)	161.6
N3−H3D···O3#4	0.89	2.05	2.808(4)	142.9
N3−H3F···O19	0.89	1.85	2.742(4)	179.8
O4−H4···O12#5	0.9	1.93	2.778(3)	157.2
O14−H14···O9#5	0.79	2.13	2.892(3)	162.2
O22−H22···O25	0.87	1.94	2.792(4)	166.5
O26−H26B···O7#2	0.85	2.03	2.819(5)	154.2
O26−H26A···O13	0.85	1.90	2.751(4)	178.4
O27−H27B···O7	0.91	1.91	2.719(4)	148.0
O27−H27A···O30	0.85	2.00	2.720(7)	141.2
O29−H29A···O1#6	0.85	1.93	2.775(4)	171.3
O30−H30B···O16#7	0.85	2.11	2.869(7)	149.2
O30−H30A···O26#7	0.85	2.10	2.889(8)	154.8

Symmetry transformations used to generate equivalent atoms:
#1 −x+1,−y+2,−z #2 −x+1,−y+2,−z+1 #3 x,y+1,z #4 x−1,y,z
#5 −x+1,−y+1,−z+1 #6 −x+2,−y+1,−z #7 x+1,y,z

Reference:

1. Wang, Y.; Li, B.; Qian, H.J.; Wu, L.X. Controlled triol−derivative bonding and decoration transformation on Cu−centered Anderson−Evans polyoxometalates. *Inorg. Chem.* **2016**, *55*, 4271−4277, doi:10.1021/acs.inorgchem.6b00008.
